# A Mobile-Based Comprehensive Weight Reduction Program for the Workplace (Health-On): Development and Pilot Study

**DOI:** 10.2196/11158

**Published:** 2019-11-04

**Authors:** Min Kyu Han, Belong Cho, Hyuktae Kwon, Ki Young Son, Hyejin Lee, Joo Kyung Lee, Jinho Park

**Affiliations:** 1 SK hynix International Medical Center Wuxi, Jiangsu China; 2 Department of Family Medicine Seoul National University College of Medicine Seoul Republic of Korea; 3 Department of Family Medicine Seoul National University Hospital Seoul Republic of Korea; 4 Department of Family Medicine Asan Medical Center Seoul Republic of Korea; 5 Department of Family Medicine Seoul National University Bundang Hospital Seongnam City Republic of Korea; 6 Department of Internal Medicine Korea University Guro Hospital Seoul Republic of Korea

**Keywords:** weight loss programs, smartphone, mobile phone, workplaces, obesity, obesity management

## Abstract

**Background:**

There is a growing interest in mobile technology for obesity management. Despite the known effectiveness of workplace-based weight loss programs, there are few studies on mobile phone–delivered interventions.

**Objective:**

This study aimed to develop and verify an integrated and personalized mobile technology–based weight control program, named Health-On, optimized for workplaces.

**Methods:**

A weight reduction algorithm was developed for calorie prescription, continuous monitoring, periodic feedback and reevaluation, goal resetting, and offline intervention with behavior-changing strategies. A total of 30 obese volunteers (body mass index ≥25 kg/m^2^) participated in the 12-week Health-On pilot program. The primary outcome was weight reduction, and secondary outcomes were improved anthropometric measures, metabolic profiles, and fat computed tomography measures, all assessed pre- and postintervention.

**Results:**

Health-On incorporated proprietary algorithms and several strategies intended to maximize adherence, using compatible online and offline interventions. The mean weight of 30 participants decreased by 5.8%, and median weight also decreased from 81.3 kg (interquartile range [IQR] 77.1-87.8) before intervention to 76.6 kg (IQR 70.8-79.5) after the 12-week intervention period (*P*<.001). The metabolic profiles and fat measures (blood pressure, glycosylated hemoglobin, total cholesterol, triglyceride, high-density lipoprotein, low-density lipoprotein, alanine aminotransferase, and visceral and subcutaneous adipose tissue; *P*<.05) also improved significantly.

**Conclusions:**

In this single-group evaluation of 30 participants before and after the Health-On program, body weight decreased and metabolic profiles and fat measures improved. Follow-up studies are needed to assess effectiveness and long-term adherence.

## Introduction

### Background

Obesity is a major global health problem [1], the cause of increased morbidity and mortality, and significant health care resources are expended on managing and preventing obesity and associated complications [2-5]. Of the options for the treatment of obesity, lifestyle interventions are foundational regardless of augmentation by drug therapy or bariatric surgery. [6,7]. Two principles are central to obesity lifestyle interventions: negative calorie balance and maintenance of it. For physicians, a clinical setting of fragmentary visits and short consultations makes individualized feedback and instruction problematic. For patients, a daily food intake or physical activity diary is intrusive and recollections are unreliable [8].

There is growing interest in lifestyle interventions using smart devices [9], and they can be effective tools to combat obesity, helping to overcome the limitations of current interventions [10,11]. They can also be helpful to achieve *comprehensive lifestyle modification* that is proved to be strongly effective to manage obesity [7,12]. Comprehensive strategy includes group intervention, online or offline interventions on diet and physical activity, frequent monitoring and feedback, and education.

Considering these things, workplace is one of the most optimal places for mobile-based comprehensive lifestyle interventions. Employee numbers will be fairly constant over the intervention period, and employer-provided resources (cafeteria and fitness center), messengers, and periodic health checkup services can be incorporated into the interventions. The features of mobile health apps (eg, real-time lifestyle monitoring and participant interactivity) can be easily integrated in workplace interventions [13]. In addition, workplace lifestyle interventions are already known to be effective for weight reduction and increased productivity and in reducing financial burden on employees [14-16]. Despite these advantages, mobile-based interventions in practice are rare.

### Objectives

Against this background, we developed a comprehensive mobile app (Health-On) and embarked on a pilot study to verify its workplace feasibility and assess the clinical outcomes before and after the implementation of this program.

## Methods

### Development of the Health-On App

**Figure 1:**
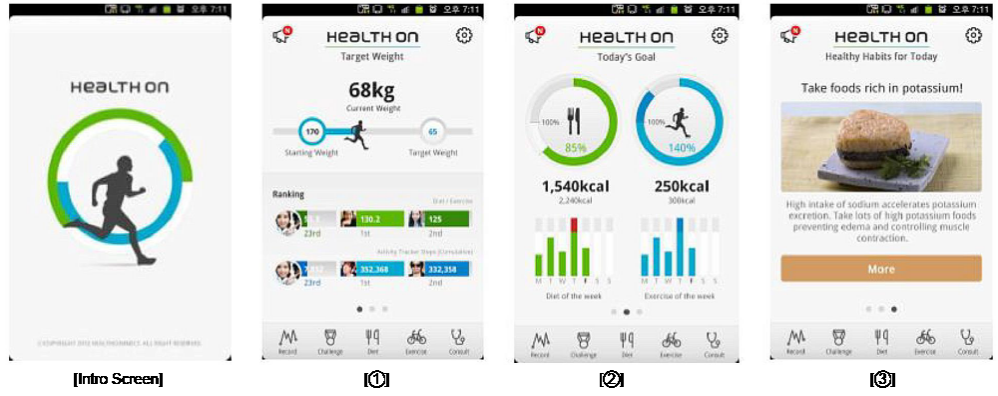
Main pages.

Health-On is a program that combines online devices (mobile app and smart watch) and offline interventional resources (cafeteria, fitness center, and peer support; Figure 1).

Researchers first developed the Health-On mobile phone app using the Android software development kit (SDK r20.0.3) [17].

To accommodate the first principle, negative calorie balance, we developed an equation to calculate total energy expenditure (TEE) and an algorithm to prescribe diet and physical activities. For the second principle, maintenance of negative calorie balance, we created a daily diet or physical activity tracker and applied behavior change strategies.

### Equation for Calculating an Estimation of Total Energy Expenditure

To identify the calorie consumption or expenditure negative balance, we must calculate TEE and maintain a TEE greater than calorie intake. TEE is obtained by summating resting metabolic rate (RMR), the thermic effect of activity (TEA), and the thermogenic effect of food (TEF). We chose the predictive Cunningham equation for measuring RMR as it is an accurate formula for estimating RMR using fat-free mass and is close to measured values in Korea [18,19]. For measuring TEA, we used the International Physical Activity Questionnaire Short Form (IPAQ-SF) [20,21]. However, IPAQ-SF does not include calories expended in daily activities, for example, showering or speaking. Thus, we estimated calorie expenditure from these to be 10% of RMR, on the premise that obese participants are sedentary [22]. TEF was assumed to be 10% of RMR + TEA [23].

TEE can be expressed as follows: TEE = RMR + TEA (1) + (2) + TEF, where (1)=kcal consumption from IPAQ-SF and (2)=kcal consumption not included in the IPAQ-SF (eg, walking <10 min and other daily activities). This equation was used to maintain the negative calorie balance between dietary intake and physical activities.

### Diet and Physical Activity Prescription Algorithm

Following accepted guidelines [7], we set a recommended goal of 7% baseline body weight loss over 12 weeks, divided into 3.0%, 2.5%, and 1.5% targets for each 4-week period. We assumed that a 7000 kcal expenditure is needed to lose 1 kg of body weight [24]. Subjects themselves determined how many negative calories they needed from their control variables, for example, how much they should cut down on eating or how much they should increase physical activity to achieve their weight reduction goals. Then, the algorithm suggested the goals of daily calories from dietary intake necessary to achieve weight loss. These goals and variables were adjusted periodically to reflect weight change during the process. To achieve safe weight reduction, we set quantitative parameters of a minimum of 1200 kcal per day for women and 1500 kcal for men [7].

### Convenient Method of Tracking Daily Diet and Physical Activity

#### Diet

Dietary intake could be entered in the app either by inputs on a pre-entered menu or by inputting using the search option.

#### Pre-Entered Inputs

We applied various methods to minimize user effort in keying in calories. First, the workplace cafeteria daily menu nutritional data were entered into the app. In addition, menus of restaurants nearby the workplace were entered. Menus that were frequently entered by app users were also pre-entered, thereby automatically appearing in the app.

#### Input by Search

We embedded the Canpro4.0 and FanTasy (Food and Nutrition Database System) databases [25,26]. When users consumed foods not on the pre-entered menu, they could search in the app. The typical Korean diet was provided in an easy-to-search select-and-enter list. Meals not on the list could be entered directly by the user.

#### Physical Activity

Physical activity could be entered in the app either by automatic input or manually.

#### Automatic Input

**Health-On enables connectivity with an activity tracker, for example, a smart watch,** to measure usual physical activity. The collected data (expended calories and step count) were automatically sent every hour to the app via Bluetooth and made available to users.

#### Manual Input

The app enables entry of type, strength, and frequency and duration of aerobic exercise, strength exercise, and common sports, as activity is difficult to measure while using fitness equipment or doing aquatic exercise. This type of user-provided information was calculated and automatically converted into expended calories based on user weight [27].

### Behavior Change Strategies

This program contains several strategies for encouraging behavior change that are effective for weight reduction [28]. We modified these strategies for mobile technology applicability (eg, individualized tailored feedback based on personal life log and ranking). We used resources available in the workplace, for example, the cafeteria and fitness center, to optimize advantages and maximize effectiveness [29].

#### Health Age

Health-On can calculate health age from basic health information. We had previously found that a Web-based health risk appraisal (factoring in health age) can be effective for ascertaining health risks and motivating lifestyle modifications [30], and we adjusted this tool for our purposes.

#### Health Information

A team of nurses, nutritionists, and exercise trainers devised educational material with diet and physical activity tips. This information was provided in the app daily to improve exercise and dietary habits and ensure effective weight loss. In addition, counseling with a nutritionist and an exercise trainer was made available through social networks.

#### Feedback on the Life Log

We developed feedback for self-monitoring and self-reflection on periodic results. Each day, calorie intake was compared with the goal, and goal achievement provided an incentive for further progress. A nutritionist provided feedback on participants’ dietary intake records. An exercise supervisor gave feedback based on comparisons between burned calories and exercise targets. Feedback was delivered via a pop-up.

#### History Query

With the history query function, users could monitor their health examination results pre- and postprogram, weekly changes in body composition and measurements, and weekly dietary and exercise performance.

#### Competition

Good-natured competition, a useful weight loss motivation method, was introduced for promoting Health-On continuous usage. Health-On automatically adds users’ friends from contact lists, enabling competition for achievement scores or step counts.

#### Ranking

The achievement score was based on the 7.0% weight loss success or failure compared with the starting weight plus proximity to monthly goals plus dietary input frequency or activity tracker usage.

#### Step Counts

The cumulative number of steps over a period, recorded on the activity tracker’s pedometer, was used to calculate rankings.

### Health-On Program Pilot Study

#### Study Population

The primary purpose of this study was to verify Health-On’s workplace feasibility and assess the clinical outcomes.

In April 2012, an announcement targeting SK telecom workers in a workplace about the recruitment of people with body mass index of more than 25 kg/m^2^ [31] who were willing to control diet and exercise for weight loss had been made on the intranet. The Institutional Review Board of Seoul National University Hospital approved this study (IRB No.: H-1204-041-405).

#### Exclusion Criteria

We excluded anyone from the recruited members (1) who had answered at least one *yes* to the questionnaires of Physical Activity Readiness Questionnaire [32], and their eligibility to this study was judged ineligible after counseling, (2) who had answered *yes* on the eating disorder survey and were judged ineligible for this study after counseling with a doctor, (3) who had systolic blood pressure (SBP) ≥160 mmHg or diastolic blood pressure (DBP) ≥100 mmHg, fasting blood sugar (FBS) ≥160 mg/dl, triglyceride ≥500 mg/dl, low-density lipoprotein-cholesterol (LDL-C) ≥190 mg/dl and judged ineligible after counseling with a doctor owing to identification of more than one abnormality, (4) who were obese and had received any pharmacological, procedural, or surgical treatment within a month, (5) who had undergone a drastic weight change (more than 10% of body weight) within a month, (6) who had suffered from or received a procedure because of severe illness such as myocardial infarction, stroke, cancer-related disorders, and hip surgery, (7) who had been suffering from thyroid disease, and (8) who were judged by the researcher as ineligible to participate. A total of 30 people were recruited for this study.

#### Measurements

Anthropometric data (height, weight, and percentage of body fat were measured by Inbody 720), fasting blood samples, and computed tomography (CT) scans were collected via questionnaires. Data were collected twice, before and after the 12-week intervention. Participants provided written informed consent, and the Institutional Review Board approved the protocol.

The laboratory tests included alanine aminotransferase (ALT), aspartate aminotransferase, creatinine, lipid profile (total cholesterol, high-density lipoprotein-cholesterol (HDL-C), and triglyceride [TG]), FBS, and baseline glycosylated hemoglobin (HbA_1c_). Lipid profiles and blood glucose tests were performed after fasting.

Abdominal adipose tissue mass was estimated using cross-sectional images obtained by a standardized and validated CT technique [33]. Participants were examined in the supine position with a 16-detector row CT scanner (Somatom Sensation 16; Siemens Medical Solutions). A single umbilicus level 5-mm slice image was obtained. The total abdominal adipose tissue area (subcutaneous adipose tissue area plus visceral adipose tissue (VAT) area was calculated using specialized software (Rapidia 2.8; Infinitt) with the attenuation values within a range of –250 to –50 Hounsfield Units. We used VAT ≥100 cm^2^ as the criterion for visceral obesity [34].

#### Intervention

On the basis of these health indicators, weight reduction goals and target net kilocalories were individually determined through the Health-On algorithm.

For the first 4 weeks, a 600 to 700 kcal breakfast was provided to create a habit of not skipping breakfast. Participants were encouraged to join Group eXercise once a week at the company gym. Participants were able to check goal achievement by taking Inbody measurements regularly.

A specialist sent review data (LifeStyle Guide) every fortnight, and personal contact was scheduled after the initial health checkup, when checking monthly goals and during a poststudy follow-up.

#### Outcomes

The primary outcome of the study was the change in weight of participants before and after the program. We also measured the change in percentage of body fat, lean body mass (kg), waist circumference (cm), serum TG, HDL-C, LDL-C, non–HDL-C, SBP and DBP (mmHg), fasting plasma glucose (mg/dL), and visceral fat as the secondary outcomes.

#### Statistics

The continuous demographic variable and the baseline variable were summarized using descriptive statistics (means with standard deviations and medians with ranges). The categorical demography characteristics were summarized by frequency distribution and percentages. Comparison of the differences between pre- and poststudy outcomes was done with a Wilcoxon signed-rank test.

All analyses were conducted using STATA version 12.1 for Windows (StatCorp), with a *P* value <.05 used to indicate statistically significant differences.

## Results

The interventions discussed in the Methods section are incorporated into the app.

### Health-On App (Online Intervention)

**Figure 2:**
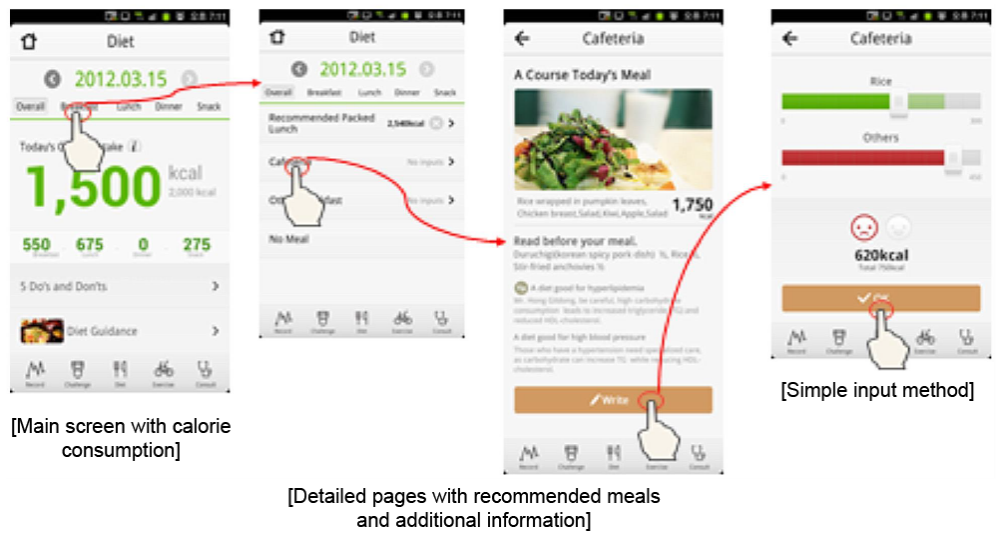
Diet pages.

**Figure 3:**
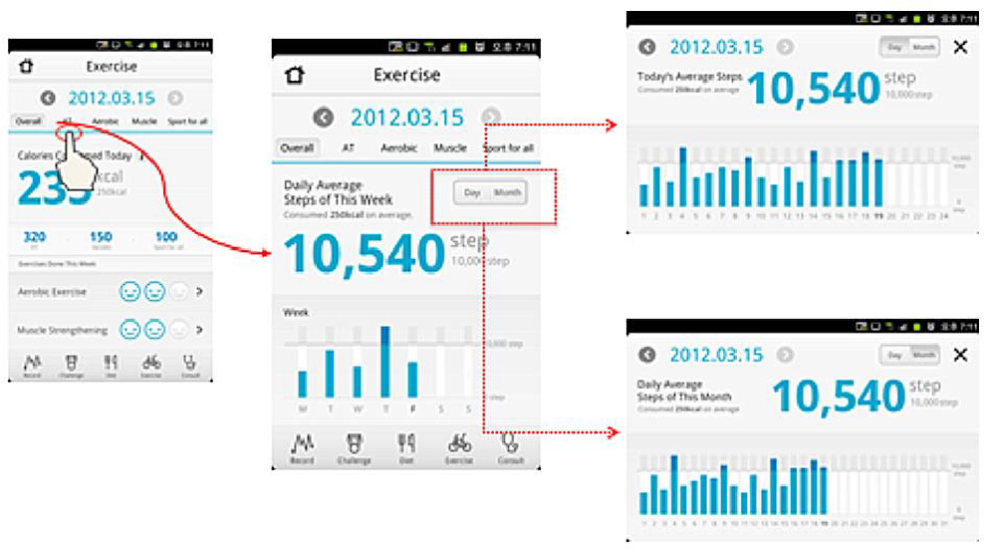
Physical activity pages: activity tracker.

**Figure 4:**
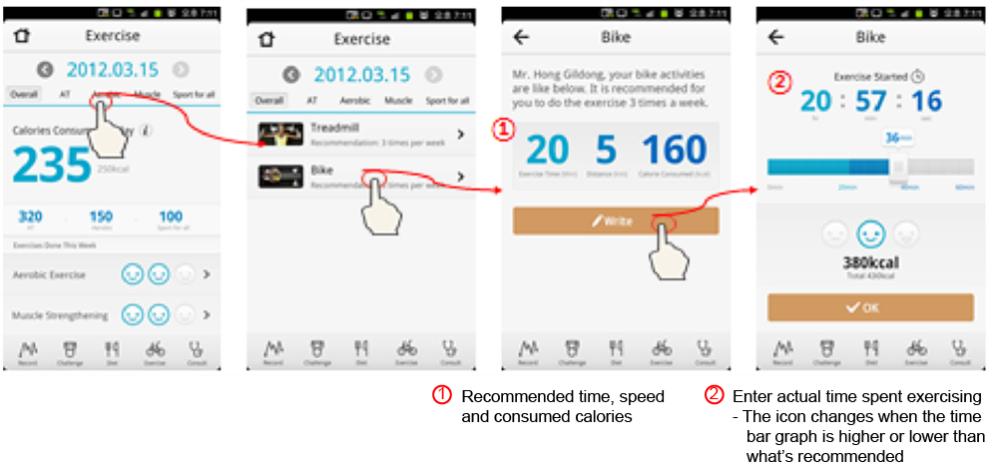
Physical activity pages: aerobic exercise.

**Figure 5:**
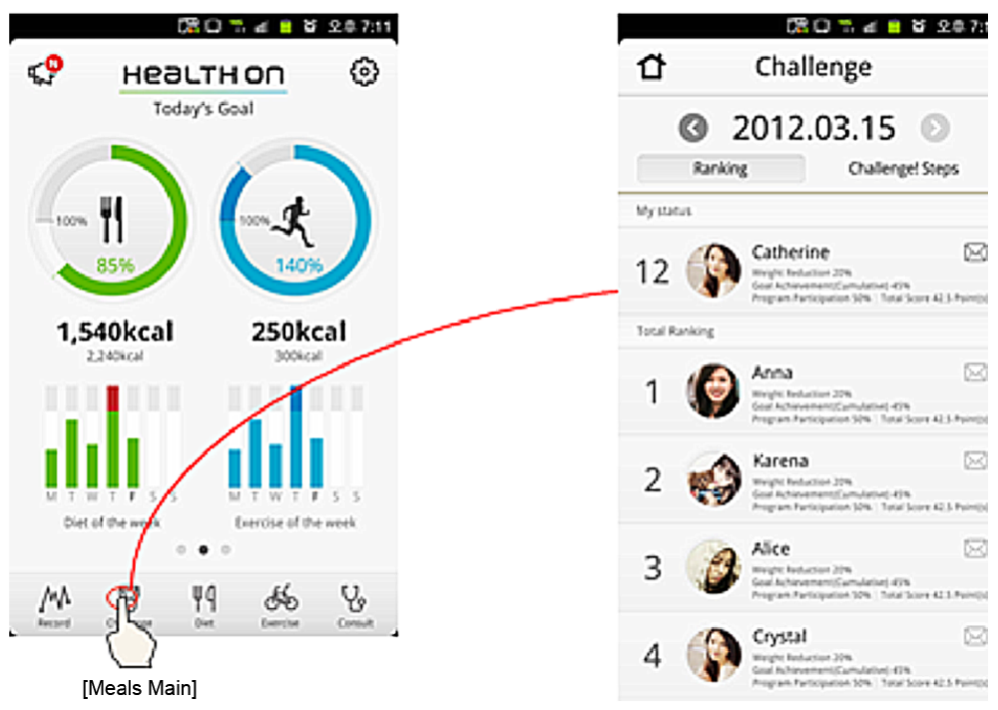
Challenge and ranking pages.

Health-On has 4 theme pages: main, diet, physical activity, and challenge and ranking. Each page allows users to easily see their achievements and to maximize user convenience and app effectiveness with a simple user interface (UI). The theme icons are on the tab menu, making movements between pages smooth and convenient (Figures 2-5).

### Offline Intervention

Recommended face-to-face components in internet-delivered weight reduction interventions are included; this increases intervention use and effectiveness [35]. Therefore, the offline intervention for Health-On enables nurses, nutritionists, and exercise trainers to inspect goal achievements and provide periodic feedback.

### Pilot Study

Table 1 shows the baseline characteristics of the participants. Median age was 39 (IQR 35-42) years, and 28 (93.3%) were male. A total of 9 people (30%) had an education level above Master’s degree, and the rest were college graduates, suggesting a relatively high level of education.

**Table 1:** Baseline characteristics of study participants (n=30).

Variable			Value
Age (years), median (IQR)			39 (35-42)
Male sex, n (%)			28.0 (93.3)
**Academic background, n (%)**			
	Bachelor’s degree	21 (70.0)	
	Graduate degree or higher	9 (30.0)	
Current smoker, n (%)			11 (36.7)
Drinking frequency per week (days), median (IQR)			1.0 (1.0-2.0)
**Anthropometry, median (IQR)**			
	Height (cm)	170.4 (166.5-173.0)	
	Weight (kg)	81.3 (77.1-87.8)	
	Waist circumference (cm)	96.8 (93.0-102.5)	
	BMI^a^ (kg/m^2^)	28.0 (27.2-30.3)	
**Bioimpedance measurement, median (IQR)**			
	Lean body mass (kg)	55.4 (51.6-58.3)	
	Body fat (%)	28.2 (25.5-30.8)	
**Metabolic profile, median (IQR)**			
	Systolic BP^b^ (mmHg)	128 (118-132)	
	Diastolic BP (mmHg)	75 (70-81)	
	Fasting plasma glucose (mg/dl)	92 (87-98)	
	HbA_1c_^c^ (mg/dl)	5.6 (5.5-5.8)	
	Total cholesterol (mg/dl)	205 (174-228)	
	Triglyceride (mg/dl)	159 (108-214)	
	HDL^d^ cholesterol (mg/dl)	45.5 (37.0-53.0)	
	LDL^e^ cholesterol (mg/dl)	131.5 (97.0-155.0)	
	Non-HDL cholesterol (mg/dl)	154 (129-195)	
	AST^f^ (IU/L)	24 (19-31)	
	ALT^g^ (IU/L)	30.5 (18-59)	
**Fat CT^h^, median (IQR)**			
	CT fat ratio	0.64 (0.50-0.94)	
	Visceral fat (mm^2^)	140.5 (110.4-192.9)	
	Subcutaneous fat (mm^2^)	224.3 (184.0-292.8)	

^a^BMI: body mass index (calculated as weight in kilograms divided by height in meters squared).

^b^BP: blood pressure.

^c^HbA_1c_: glycosylated hemoglobin.

^d^HDL: high-density lipoprotein.

^e^LDL: low-density lipoprotein.

^f^AST: aspartate aminotransferase.

^g^ALT: alanine aminotransferase.

^h^CT: computed tomography.

The median of variables was weight 81.3 kg (IQR 77.1-87.7), abdominal circumference 96.3 cm (IQR 93.0-102.5), and BMI 28.0 (IQR 27.2-30.3).

Visceral fat and subcutaneous fat were measured by the fat CT, with the median being 140.5 cm^2^ (IQR 110.4-192.9) and 224.3 cm^2^ (IQR 184.0-292.8), respectively.

Changes in anthropometric and metabolic profiles between pre- and postintervention are shown in Table 2. The mean body weight was decreased by 5.8%. The median of weight, waist circumference, BMI, lean body mass, and body fat percentage reduced significantly, as did most of the metabolic profiles, especially HbA_1c_ and non–HDL-C. The changes in visceral fat and subcutaneous fat were statistically significant.

**Table 2:** Comparison of outcomes before and after Health-On program (n=30).

Outcomes		Baseline, median (IQR)	Final, median (IQR)	Difference, median (IQR)	*P* value^a^
**Anthropometric measurement**					
	Weight (kg)	81.3 (77.1 to 87.8)	76.6 (70.8 to 79.5)	–6.2 (–8.4 to 3.9)	<.001
	Waist circumference (cm)	96.8 (93.0 to 102.5)	88 (84.5 to 95.0)	–9.2 (–11 to 5.5)	<.001
	BMI^b^ (kg/m^2^)	28.0 (27.2 to 30.3)	25.7 (24.6 to 28.0)	–2.2 (–3.4 to 1.5)	<.001
**Bioimpedance measurement**					
	LBM^c^ (kg)	55.4 (51.6 to 58.3)	54.0 (51.2 to 57.1)	–1.0 (–2.1 to 0.3)	.003
	Body fat (%)	28.2 (25.5 to 30.8)	24.1 (20.9 to 27.6)	–4.65 (–6.5 to 1.8)	<.001
**Metabolic profile**					
	SBP^d^ (mmHg)	128 (118 to 132)	121 (107 to 127)	–5.5 (–14 to 2)	.002
	DBP^e^ (mmHg)	75 (70 to 81)	70 (63 to 82)	–4 (–10 to 2)	.08
	Fasting plasma glucose (mg/dl)	92 (87 to 98)	90 (86 to 99)	–1.5 (–8 to 3)	.38
	HbA_1c_^f^ (mg/dl)	5.6 (5.5 to 5.8)	5.4 (5.2 to 5.6)	–0.2 (–0.4 to 0.1)	<.001
	Total cholesterol (mg/dl)	205 (174 to 228)	185 (158 to 198)	–15.5 (–31 to 7)	.001
	Triglyceride (mg/dl)	159 (108 to 214)	89 (57 to 124)	-63 (–113 to 25)	<.001
	HDL^g^ cholesterol (mg/dl)	45.5 (37.0 to 53.0)	51 (43 to 62)	6.5 (1 to 10)	.007
	LDL^h^ cholesterol (mg/dl)	131.5 (97.0 to 155.0)	107.5 (88 to 125)	–17.5 (–31 to 3)	.001
	Non-HDL cholesterol (mg/dl)	154 (129 to 195)	126.5 (107 to 145)	–21.5 (–36 to 11)	<.001
	AST^i^ (IU/L)	24 (19 to 31)	22.5 (17 to 32)	–1.5 (–9 to 4)	.23
	ALT^j^ (IU/L)	30.5 (18 to 59)	20.5 (16 to 30)	–5 (–28 to 1)	.006
**Fat CT^k^**					
	CT fat ratio	0.64 (0.50 to 0.94)	0.63 (0.4 to 0.92)	–0.05 (–0.19 to 0.06)	.09
	Visceral fat (cm^2^)	140.5 (110.4 to 192.9)	95.6 (732.4 to 133.0)	–39.4 (–60.3 to 16.3)	<.001
	Subcutaneous fat (cm^2^)	224.3 (184.0 to 292.8)	165.0 (117.3 to 237.9)	–56.4 (–77.0 to 20.6)	<.001

^a^Wilcoxon signed-rank test.

^b^BMI: Body Mass Index (calculated as weight in kilograms divided by height in meters squared).

^c^LBM: lean body mass.

^d^SBP: systolic blood pressure.

^e^DBP: diastolic blood pressure.

^f^HbA_1c_: glycosylated hemoglobin.

^g^HDL: high-density lipoprotein.

^h^LDL: low-density lipoprotein.

^i^AST: aspartate aminotransferase.

^j^ALT: alanine aminotransferase.

^k^CT: computed tomography.

## Discussion

### Principal Findings

The purpose of this study was to develop and verify a comprehensive mobile-based workplace weight reduction program.

Health-On has several strengths compared with other weight reduction apps. Although there are more than 10,000 such apps, a recent review reported that many have insufficient evidence-based content vis-a-vis US government diet and exercise recommendations [35,36]. Health-On incorporates a scientifically evidenced algorithm for estimating negative calorie balance and behavioral strategies established as effective for weight management. In addition, requiring people to complete preprogram health questionnaires, enabled avoidance of participants with health risks. Therefore, Health-On can minimize possible adverse effects, often neglected by other apps.

We created a more convenient method of keying in data. As noted in previous self-monitoring studies, the less intrusive the tool, the higher the rate of adherence [37]. We devised several convenient ways to improve user adherence by collecting data on dietary intake and physical activities. The accuracy and sustainability of keeping a food diary is important; therefore, we attempted to create a user-friendly and nonintrusive UI. Instead of the food diary method, we installed a volume bar for pre-entered diet fields so people can more conveniently record food consumption. This significantly relieves the onerous task of name searching and certainly increases adherence to and sustainability of the program compared with a paper food diary. As for monitoring physical activities, the entry method is more convenient than the paper diary technique that most other apps require. A better approach, such as activity tracking via wearable devices and auto-synchronizing data via Bluetooth, can improve program adherence and compensate for participant self-reporting limitations. Furthermore, physical activities can be quantified and evaluated more accurately.

This program incorporated clinically proven effective behavior change strategies for facilitating weight loss, changes in diet and exercise, and preventing relapse [24,28,37,38]. It is known that if, when deciding the target weight loss, users’ diet-exercise preferences are reflected on the target, it will result in greater success [39]. Furthermore, Health-On enables patients to monitor their lifestyles through an everyday life log and allows professionals to provide feedback and educational information. Behavioral changes are promoted through self-monitoring, education, feedback, and competition.

This comprehensive approach combines interventional components that have the strongest effect on obesity (eg, healthy meals) and workplace wellness [15,40]. Most apps do not integrate diet, physical activity, and behavior change strategies [15,41].

Workplace weight management is a highly effective approach to intervention [3,7,16], and Health-On is one of the mobile-based workplace interventions that maximize the advantages and suitability of workplace lifestyle interventions. Effective population-based weight management is feasible in workplaces, from which supplementary benefits flow, for example, increased productivity, lower absenteeism, and reduced medical costs [3,7,15,16].

In a meta-analytic review, Verweij et al [42] analyzed the effectiveness of workplace interventions targeting physical activity and/or dietary behavior on outcomes. Their study delivered moderate quality evidence that workplace physical activity and dietary behavior interventions significantly reduce body weight (9 studies; mean difference -1.19 kg [95% CI –1.64 to –0.74]) [43]. These studies did not use mobile interventions. Stephans et al [43] performed a 3-month randomized controlled trial using a behavior-based mobile phone app. They reported that the difference in weight change between groups was statistically significant (mobile phone group -1.8 kg vs Control group +0.3kg; *P*=.03). Direct comparison is difficult owing to research methodological differences, but the weight loss of Health-On is considerable. Changes in anthropometry, bioimpedance measures, metabolic profile, and visceral fat were also significant [42].

During calorie restriction, muscle wasting prevention is important because muscles play an important role in improving metabolic profiles and reducing insulin resistance [44]. Nevertheless, without the simultaneous modification of diet and physical activity, losing weight with a low-calorie diet alone might reduce fat and also fat-free mass [45]. It may superficially seem like a significant reduction in weight but resisting metabolic rate and insulin sensitivity would become lower because of a reduction in muscle mass, thus resulting in the tendency of weight regain [45]. This study was characterized with a body fat reduction –4.65% median difference (MD; IQR –6.5 to –1.8), and a decrease in muscle mass 1.0 kg MD (IQR –2.1 to –0.3). By preventing muscle loss that could generally occur with a lower calorie diet, it could be concluded that there had been an ideal weight loss achievement with decreased tendency to long-term weight regain.

Interestingly, there was a significant reduction in visceral fat levels despite the short intervention period. Accumulation of VAT is a clinically important marker as it increases insulin resistance to induce metabolic syndrome and heightens cardiovascular risks; therefore, the results of this study demonstrate that Health-On could be effective in managing obesity and lowering the cardiovascular disease risks [46,47].

Through the reduction in obesity rates in workers, as many studies’ results previously showed, improved productivity, and reduced absent rates, a reduction in expenses could be expected from the employer [48-50].

### Limitations

This study had several limitations:

1. This study was designed as a pilot test so that direct weight reduction effect of Health-On could be measured. This design is limited in that it does not allow direct comparisons with other forms of treatment. Prospective, randomized trials with appropriate controls are needed.
2. Health-On was developed on an energy balance equation, which was based on scientific evidence such as Cunningham’s equation, which estimates RMR indirectly [51]. Metabolic rate and calorie expenditure are assumed, not determined through individual differences such as genetic susceptibility to obesity.
3. IPAQ-SF is the desired instrument to measure physical activities, but it considers those within the previous 7 days. There can be differences between daily calorie expenditure calculated from IPAQ-SF and the actual level of physical activity. Although we attempted to compensate for these shortcomings with activity trackers, discrepancies could occur [52].

Despite the short period of this Health-On study, weight reduction was substantial. However, the persistence of weight reduction was unpredictable. Thus, further research for postprogram body weight changes and management is indicated.

### Conclusions

Health-On is a promising workplace intervention tool that can be used in similar environments, for example, universities and the military, with minimal modifications. The results of this study could form a base for designing randomized clinical trials for comparison with conventional weight loss programs. Henceforth, future research should focus on the additional benefits and longitudinal effects of this program.

## Abbreviations

ALT: alanine aminotransferase
CT: computed tomography
DBP: diastolic blood pressure
FBS: fasting blood sugar
HDL-C: high-density lipoprotein-cholesterol
IPAQ-SF: International Physical Activity Questionnaire Short Form
IQR: interquartile range
LDL-C: low-density lipoprotein-cholesterol
MD: median difference
RMR: resting metabolic rate
SBP: systolic blood pressure
TEA: thermic effect of activity
TEE: total energy expenditure
TG: triglyceride
UI: user interface
VAT: visceral adipose tissue
